# A Model of Triage of Serious Spinal Pathologies and Therapeutic Options Based on a Delphi Study

**DOI:** 10.3390/medicina59071283

**Published:** 2023-07-11

**Authors:** Philippe Meidinger, Amélie Kéchichian, Nicolas Pinsault

**Affiliations:** TIMC-IMAG UMR CNRS 5525, ThEMAS Team, Université Grenoble Alpes, Domaine de la Merci, 5 Avenue du Grand Sablon, 38700 La Tronche, France

**Keywords:** red flags, clinical reasoning, back pain, cauda equina syndrome, spinal fracture, spinal infection, malignancy, serious pathology, spinal pain, low back pain, diagnostic triage

## Abstract

*Background and Objectives*: The relevance of red flags in serious spinal pathology (SSP) has evolved throughout the last years. Recently, new considerations have been proposed to expand the consideration of red flags. The purpose of this study was to determine, approve and test a model for the triage and management process of SSPs based on the latest data available in the literature. *Materials and Methods*: The SSP model was initially built on the basis of a literature review. The model was further determined and approved by an expert panel using a Delphi process. Finally, clinical scenarios were used to test the applicability of the model. *Results*: After three rounds of the Delphi process, panellists reached a consensus on a final version of the model. The use of clinical scenarios by experts brought about reflexive elements both for the determined model and for the SSPs depicted in the clinical cases. *Conclusions*: The validation of the model and its implementation in the clinical field could help assess the skills of first-line practitioners managing spinal pain patients. To this end, the development of additional clinical scenarios fitting the determined model should be further considered.

## 1. Introduction

In several countries, patients can access physiotherapy care without being referred by a physician [1,2]. In some other countries, patients are referred to physiotherapists by family physicians. In these countries, new models of care integrating a more autonomous role of the physiotherapist are being gradually implemented. For instance, new models of task sharing and shifting are being implemented in France between general practitioners and physiotherapists for patients with non-urgent musculoskeletal disorders, such as ankle sprains or low back pain [3]. Its implementation is a major shift for the evolution of physiotherapists’ skills, roles and responsibilities, since French patients are traditionally referred to physiotherapists by physicians.

Within the framework of the above-mentioned model of care, an interprofessional training is planned in order to consolidate the knowledge of physiotherapists on triage and prescribing medication and sick leave certificates. This is the first step towards the extension of French physiotherapists’ roles and responsibilities. The development of this model emphasizes the need for French physiotherapists to acquire adequate skills to manage patients as first-contact primary care practitioners. The development of self-confidence in their ability to conduct clinical reasoning, triage and differential diagnosis process is required. The early identification of serious spinal pathologies (SPPs) among patients with low back pain should especially be part of their clinical practice as first-contact practitioners.

Four SPPs have been identified by authors [4,5]: fracture, cancer, infection and cauda equina syndrome (CES). Some authors also included axial spondyloarthritis [6,7]. All these SSPs are relatively rare, as their prevalence does not exceed 1% in primary care [8]. Nevertheless, these pathologies can have a serious impact on the patient’s health status and lead to significant costs [9]. Thus, systematic screening for the early identification of SSP signs and symptoms should be performed by first-contact practitioners.

SSP triage would potentially allow a decrease in the loss of chances for patients with the earlier identification of serious pathologies. The evolution of physiotherapists’ skills to manage patients as primary care practitioners could lead to a decrease in general practitioners’ workload [10]. It could also reduce emergency department use [11]. However, this evolution has to be implemented with caution to ensure patient safety. Physiotherapists should have the necessary skills for both the triage and subsequent management of patients with low back pain.

To help clinicians in the triage of SSPs, several guidelines support the use of the red-flag concept [12,13,14]. Red flags are signs and symptoms that potentially help in the detection of the so-called serious pathologies [4]. However, the red flags in these guidelines suffer from a lack of standardization and poor diagnostic capacity [14]. Among the large variety of red flags described in the literature, only a few of them seem to be relevant [13,14,15,16]. These red flags did not enable clinicians to exclude or identify SSPs based on the positive and negative likelihood [15]. Furthermore, given the prevalence of these conditions and the clinimetry of red flags, the post-test probability of having a serious spinal condition was relatively low [17]. Several authors have called for abandoning discussions because there is currently no consensus on the definition of red flags [16]. Other authors suggested finding a standard definition [15]. Finally, low-quality study methodologies do not provide a high evidence level about red flag use. Larger sample sizes in primary care settings are needed [17,18]. Investigating the association between clusters of several red flags in order to increase their diagnostic capacities may also be an encouraging future research area [15,17,18]. Studies should be conducted with higher quality and larger sample sizes. The development of a model for the triage of spinal pain patients based on the highest level of evidence and experts’ opinions could help primary-contact practitioners to acquire adequate skills for the assessment and management of these patients.

Thus, the purpose of this study was to determine and approve a model for the triage and management process of SSPs based on the latest data available in the literature. The secondary objective was to test the applicability of the determined model through the use of clinical scenarios.

## 2. Materials and Methods

A Delphi study was conducted based on available and relevant evidence regarding the triage of patients with serious spinal pathologies. Two steps were undertaken: the first one aimed to synthetize the available literature on the topic through a literature review, and the second one consisted of Delphi process development.

### 2.1. Step 1: Literature Review and Model Constitution

A literature review of red and yellow flags was conducted in order to gather the latest data available in the literature on this subject. The research was conducted using the following 4 databases: Medline, Cochrane Library, Embase and Google Scholar; the period studied was from 2015 until March 2021. The model was constituted following systematic bibliographic research. These search terms were extended with specific terminology and synonyms using Boolean operators, and the respective Medical Subject Headings are reported in Appendix A. Some specific filters or limits were used; for example, only English articles were included. The selected studies were then used to create a model that included triage and therapeutic options to manage patients with low back pain. The model evaluated in this study was created using diagram.net and can be found in Appendix B for each round. The created model was then tested and modified through a Delphi process.

### 2.2. Step 2: Delphi Process

This section reports recent recommendations about the Delphi process [19,20,21]. The Delphi methodology was conducted because of the relatively weak and conflicting literature on the subject of red flag use and the triage of spinal pain patients. Following the current literature, the “panellist” term was preferred to “experts” [22,23].

#### 2.2.1. Inclusion Criteria for the Participants

The following inclusion criteria were used for the selection of panellist participants:

- A minimum of 5 years post-graduation in medicine (general practitioner, rheumatologist, emergency physician) or physiotherapy;
- Clinical practice including a strong musculoskeletal focus (especially on low back pain) associated with knowledge on the issues addressed by the model (triage, management of low back pain);
- Or research activity/publications related to the triage and management of musculoskeletal disorders;
- Fluency in speaking and and reading the French language (since the model was designed in French).

#### 2.2.2. Consensus Definition

We used single-choice questions for the majority of the questions, from 1 (insufficient) to 7 (sufficient)-point Likert-scale response options (ordinal variable) [21]. We chose to start with a central tendency within a specific range (unrestricted) for more readability [19]. We took the medians rather than the averages to avoid the influence of extreme values, especially considering the number of panellists included. An item or group of items was considered to be suitable if the median was greater than or equal to 5/7. An item or group of items obtaining a median between more than 3/7 and less than 5/7 was considered subject to discussion. For a median of less than 3/7, the item or set of items was considered not validated. The assignment of a score was mandatory. In addition, the participants were also asked to comment on any of their scores (for the first round). For the second and third rounds, the participants were asked to comment and offer suggestions when the scores were below 5/7. In the first round, the comments were used as a reference to find a common direction for the model. For the second, if questions were specified in the comments, the participants were independently contacted to answer them. Three rounds for this modified-Delphi model were set a priori, considered the stopping point if consensus was not reached before.

#### 2.2.3. Contents of the Panellist Questionnaire

The *LimeSurvey* (*GmbH Hamburg*, *Germany*) platform was used to administer the survey. This platform was secured by the encryption of data. Information about the objectives of the study, the construction of the model, the duration of the questionnaire (average duration of 25 min), the time allowed for its completion (3–4-week delay) and the scoring system were first provided to participants. The questionnaire was anonymous. Each participant received a personalized email that contained a link to the questionnaire, the model (modified for rounds 2 and 3), the bibliography that was used (updated between the rounds), an explanatory summary (for rounds 2 and 3) and a mind map (for rounds 2 and 3). The mind map provided a graphical representation of the participants’ results (medians and interquartile ranges were indicated for each answer). The questionnaire contained four groups of questions based on the relevance (interest and localization of the items/groupings of items of the model, the formulation of the items/groupings of items of the model, clinical reasoning through this model, the model’s global approval (qualitative data) and information about the participants.

The question on the assessment of clinical reasoning through the model was as follows: Does the clinical reasoning, associated with reading the algorithm, seem relevant to you? For round 1, the following question about the model’s global approval was asked: Does the algorithm cover all possible scenarios concerning the triage of serious spinal pathologies and the management of low back pain? For round 2, the question was as follows: In your opinion, is the proposed synthesis satisfactory to consider the triage and management of spinal pathologies?

### 2.3. Step 3: Model Applicability through Clinical Scenario

In the third and final rounds, the panellists were asked to approve the latest version of the model. The panellists who approved the model were asked to test it through five clinical scenarios, which are presented in Appendix C. The participants’ level of concern, degree of confidence regarding the diagnosis and the clinical evidence for the diagnosis were asked using the approved model. The first clinical scenario was related to a suspicion of a fracture. The second clinical scenario was related to the probable presence of ankylosing spondylitis. The third one applied to the probable presence of cancer. The fourth one applied to the probable presence of cauda equina syndrome. The fifth clinical scenario was related to low back pain without any serious pathologies. The goal of this last scenario was to explore whether the panellists would have considered the nervous system examination (see the change in the last question). The main objectives of these clinical cases were:

- To explore the applicability of the model in clinical practice through the clinical reasoning process of the panellist;
- To build clinical scenarios for research and educational purposes.

## 3. Results

### 3.1. Literature Review

After the article selection, 27 studies were included based on full-text selection. The whole bibliography used to create the model is available in the references (4–18). The studies were used to create the triage model to be approved by the panellists, as described in the following section. The initial version of the created model is available in Appendix B.

### 3.2. Delphi Study

#### 3.2.1. First Round

The sample targeted a panel of 11 panellists. The panel was composed of five physicians and six physiotherapists in order to include a varied range of practitioners who primarily manage patients with spinal pathologies [21,22,23]. Among the physicians, general practitioners, emergency physicians, physical rehabilitation physicians and rheumatologists were represented. French-speaking physiotherapists were recruited from different countries: Canada, Belgium, Switzerland and France. Information on the panellists is reported in Table 1.

Out of the eleven panellists initially targeted, nine agreed to participate. At the end of the first round, only six of them fully completed the questionnaire. The panel was composed of five physiotherapists and one general practitioner. Of the three panellists who agreed to participate but did not complete the first round, two panellists did not answer the questionnaire in the first round, and one panellist partially completed the questionnaire.

The following results regarding the *relevance* of the model’s items at the end of the first and second rounds were found:

- Three groupings of elements were validated by the panellists: the notion of trauma, the medical history and the physical examination;
- In this same category, five groupings of elements were subject to discussion according to the panellists: fracture, infection, ankylosing spondylitis, cauda equina syndrome and response;
- Five groupings of elements were not approved by the panellists: history, cancer, flags (yellow, blue and black), treatment and additional investigations.

The following results regarding the formulation of the model’s items at the end of the first and second rounds were found:

- Seven element groupings were validated by the panellists: the notion of trauma, physical examination, fracture, cancer, cauda equina syndrome and treatment;
- Six groupings of elements were subject to discussion according to the panellists: history, infection, ankylosing spondylitis, flags, additional investigations and favourable patient evolution;
- Finally, only one grouping of elements was not validated by the panellists: ankylosing spondylitis.

The results regarding the relevance and formulation of the model’s items are presented in Table 2.

The item “evaluation of the clinical reasoning in the model” is presented in Table 3 as an item needed to be further discussed. For the model’s global approval, three authors reported that there were no obvious missing elements in the model. Two authors stated that the model did not enable the specification of an urgency rating and therefore had a mixed opinion. The last one mentioned that this model did not consider any suspicions of myelopathy, vascular pathology and/or other organic pathologies. Several authors have expressed a desire to see elements of the international framework for red flags [4].

#### 3.2.2. Second Round

The model used for this second round is available in Appendix B. Regarding the relevance and formulation of this model, reported in Table 2, six groupings of elements were validated by the panellists: the notion of trauma, fracture, ankylosing spondylitis, cauda equina syndrome and level of preoccupation and decision. Within this same category, two other groups of elements were discussed by the panelists: cancer and infection. The clinical reasoning item presented in Table 3 needed to be further discussed. For the model’s global approval, four panellists answered that this model could still be improved. The last two panellists were satisfied with this model.

#### 3.2.3. Third Round

Following the results of the second round, a new version of the model was designed. This last version of the model is available in Appendix B. Five panellists out of six finally approved the last version of the model. The panellists were invited to test it through five clinical scenarios (see Section 2). The clinical scenarios used for model testing are presented in Table 4. For clinical scenario number 1, the panellists agreed to assign a high level of concern. The clinical elements that were most important for this scenario, according to the panellists, were the age of the patient, the context of the fall/trauma, being menopausal, being a smoker and being female. The most frequently suggested diagnosis was a fracture. Four panellists considered the clinical elements to be insufficient. The last panellist stated that he had sufficient clinical elements for this clinical scenario, as can be found in Appendix D.

For clinical scenario number 2, three panellists assigned a high level of concern. The two others assigned no level of concern and a moderate level of concern. The clinical elements that were most important for SpA, according to the panellists, were the age of the patient, night pain, the efficacy of nonsteroidal anti-inflammatory drugs and the presence of enthesitis. The most probable suggested diagnoses were SpA, chronic nonspecific low back pain and nonspecific mechanical low back pain. As for the clinical elements presented in the clinical scenario, four panellists stated that they had sufficient clinical elements about this clinical scenario (including two panellists who made a diagnosis of SpA and two panellists who attributed the symptoms to the other two diagnoses). One of the panellists who made the diagnosis of SpA felt that he did not have enough clinical evidence.

For clinical scenario number 3, three panellists assigned a high level of concern. The other two each assigned a moderate level of concern. The clinical elements that were most important for identifying cancer, according to the panellists, were a history of cancer, night pain, worsening of pain and thoracic irradiation. The most probable suggested diagnoses were cancer and nonspecific low back pain. As for the clinical elements presented in the clinical scenario, four panellists stated that they had insufficient clinical elements (all of them made the diagnosis of cancer). The panellist who specified that he had enough clinical evidence made the diagnosis of nonspecific low back pain.

For clinical scenario number 4, three panellists assigned a high level of concern. The other two each assigned a moderate level of concern. The clinical elements that were most important for CES, according to the panellists, were a weird sensation when the patient urinates, episodes of numbness and tingling and the presence of neurological disorders in both legs. The most probable suggested diagnoses were CES and mechanical low back pain. As for the clinical elements presented in the clinical scenario, four panellists stated that they had insufficient clinical elements about this clinical scenario. The panellist who specified that he had enough clinical evidence made the diagnosis of CES.

For clinical scenario number 5, three panellists assigned a moderate level of concern. The other two panellists assigned no level of concern. The clinical elements that were most important for this scenario, according to the panellists, were no red flags, many blue and yellow flags, sedentary, stressed, divorced and the presence of tingling and pins and needles. The most probable suggested diagnoses were NSLBP and NSLBP with radicular symptoms. As for the clinical elements presented in the clinical scenario, three panellists stated that they had insufficient clinical elements about this clinical scenario. The other two panellists indicated that they had sufficient clinical evidence.

## 4. Discussion

### 4.1. Overall Results

#### 4.1.1. Findings

This study aimed at developing and testing a model for the triage and management process of SSPs. To our knowledge, such a model has not yet been developed. Regarding the final model resulting from the three rounds of the Delphi process, some elements seem to be in line with Finucane et al.’s framework [4]. At first, one of the main findings is the use of the “level of concern” notion in the clinical reasoning process mapped out by the model. Some items of the model were consistent with other studies on this topic [5,6,7,12,13,14,17,18,24,25,26,27]. Then, using clinical scenarios enabled an understanding of the panellists’ reasoning heuristic of SSP triage. Concerning the last version of the model, disagreements were found between panellists regarding the use of yellow, blue and black flags in the model. One panellist invalidated the model at the third round, explaining that flags cannot be taken into consideration for a screening. Two panellists expressed the lack of relevance of the use of yellow, blue and black flags, while the rest of the panellists underlined their importance. It seems important to point out that during their consultations, physiotherapists collect large amounts of information from their patients, which, in turn, will dynamically influence the level of concern. Regarding a zero level of concern, it is appropriate to consider the other flag categories [28]. This would result in the integration of biological factors first and then the consideration of psychosocial factors. It could lead to segmenting the biopsychosocial model (mBPS), whereas the biopsychosocial model is a whole and nonsegmentable model [29,30,31,32]. Considering red flags alone is insufficient since other factors, such as symptom progression, comorbidities, prevalence of pathology, etc., must be considered (see Appendix B). However, knowing which red flags are found in SSPs remains unavoidable.

Regarding the use of the five clinical scenarios, only the first one obtained the same level of concern from all the panellists. Four scenarios were subject to heterogeneous responses from the panellists. This can be explained by the difficulty in creating indicative clinical scenarios for SSPs. It appears that most panellists suspected an SSP when the clinical scenarios were built in this way. From the last clinical scenario, it appears that understanding and carrying out an exhaustive neurological examination should be more questioned. This crucial point should be considered when managing SSPs and other pathologies (narrow lumbar canal, neuralgia, neuropathy, neuropathic pain). Many papers encourage clinicians to perform acute neurological examinations, using the development of clinical sensory tests as an example [33,34,35,36,37].

#### 4.1.2. Delphi and Clinical Scenario Processes

Throughout the Delphi process, we noted a discrepancy between the information sent to the panellists to answer their comments and the answers proposed in the next round. In order to limit this discrepancy as much as possible, individual discussions by email were conducted with the panellists to clarify certain answers. The issue of panellists’ involvement is a relatively common topic in papers related to the consensus method [19,20,21]. It seems, therefore, appropriate to emphasize the strategy developed here in order to remain fully involved in a consensus search and obtain a plurality of opinions. Throughout these different rounds, it appears that we were able to manage and respect the opinions of each panellist in reaching the current model. The model varied significantly between the first and second rounds. This can be explained by the difficulties experienced by the panellists with answer modalities; therapeutic options can be found in Appendix B. The panellists suggested using the international framework for potential SSP red flags [4]. This significant change enables the consideration of a wider range of answers and therapeutic options. Moreover, in the model of round 2, the mention of medical history, anamnesis and physical examination to facilitate the reading of the model no longer appeared. The complexity of the first model provided a dichotomous tree structure, which had to be reconsidered following the feedback from the panellists. Furthermore, in view of the panellists’ responses, it was decided to merge the questions on wording and relevance, as shown in Table 2. This choice was made in order to avoid repetition, improve data processing and reduce the time needed to complete the survey. We noted that the items for cancer suspicion were quite different between the first and second models. Indeed, the panellists added several items for the cancer SSP in addition to the type of cancer (<5 years) and unexplained weight loss. These proposals do not seem to be consistent with the available data [4,5,24,26]. Minor changes between round 2 and round 3 are reported in Table 3 and Appendix B. Following the panellists’ remarks, changes were made to the “cancer” and “infection” items, and some clarifications in the wording have been added. Regarding the clinical cases, despite the involvement of the panellists in the validation of the model, there were divergences in the level of concern, the diagnoses and the percentage of certainty suggested. Only the first clinical case obtained the consensus of the whole panel, as mentioned in Table 4. The third, fourth and fifth clinical cases obtained a consensus of four out of five panellists for the SSP initially considered for each case. For the second case, three panellists agreed on the SSP initially envisaged in the case. In each of the clinical cases, the panellists wanted more information in order to increase their suspicion of an SSP, as reported in Appendix D. The disagreements between the panellists can be explained by the clinical cases themselves. They were intended to be concise and did not include all the elements of each SSP found in the final model.

### 4.2. Limitations, Strengths and Future Directions

The first limitation of this study is related to the literature review process. We started by analysing the latest existing data on the topic related to the model. One may regret the absence of a systematic approach and extension of the research until 2023. We may have overlooked one or several articles. Nevertheless, considering the feedback from the panellists, no shortcomings were noted. The end of the third round took place in September 2022. The second limitation of the study relates to the panellists not including specialized physicians such as a rheumatologist, an emergency physician or a physician in physical medicine and rehabilitation. Although we expected to include them in a targeted panel of six physiotherapists and five physicians, we did not reach this objective, as some panellists who were contacted did not respond to our solicitation. Only six panellists agreed to participate, including five physiotherapists and one family physician. This can be explained by the lack of time that physicians have to respond to such a study during the COVID crisis. One panellist also reported that the first version of the model was difficult to read. It can be speculated that the complexity of the first model and the length of the first questionnaire may have limited inclusion. We could have extended the recruitment period to include more physicians. However, we made the choice to respect the timeline we had previously defined. The aim was to keep the panellists involved, as they were willing to participate in the study. The clinical cases could have benefited from a more exhaustive presentation by including, for example, the results of imaging or analysis. In addition, it would have been desirable to know the strategy they could have implemented in each case. Through these clinical cases and the panellists’ responses, we were able to capture elements of clinical reasoning and improvements in the suspicion of SSP. The clinical cases proposed to the panellists were not intended to evoke a high level of concern, a total consensus or 100% certainty. This highlights that it could be interesting to develop many clinical scenarios and have them tested by physiotherapists in order to further test and develop their clinical reasoning process in SSP-specific situations. In addition, it would be interesting to take into account the uncertainty of practitioners in the context of serious spinal pathologies [38,39]. This model provides a global insight into elements to consider for SSPs. However, this model does not lessen the complexity or the uncertainty faced in the clinic. Future studies are needed to further assess the reproducibility of our model through rigorous methodology.

## 5. Conclusions

Overall, the clinical scenarios proposed here could be used to assess the skills of front-line therapists caring for patients with spinal pathologies. However, we do not yet have a weighting of the clinical elements that can define levels of concern. The development of future clinical scenarios evaluating this last point for SSPs in collaboration with panellists should be considered. In the future, the model could also be used for educational and research purposes. It could be a promising tool to improve the triage and management process of patients with low back pain and support the need for physiotherapists to acquire adequate skills to manage patients as first-contact primary care practitioners.

## Figures and Tables

**Table 1:** Characteristics of targeted panellists.

Panellists	Location	Profession and Care Access	Included
Panellist 1	France	Physiotherapist with no primary contact	Yes
Panellist 2	Canada	Primary-contact physiotherapist	Yes
Panellist 3	Switzerland	Physiotherapist with no primary contact	Yes
Panellist 4	Belgium	Physiotherapist with no primary contact	Yes
Panellist 5	France	Physiotherapist with no primary contact	Yes
Panellist 6	France	Family physician with primary contact	Yes
Panellist 7	Canada	Primary-contact physiotherapist	No
Panellist 8	France	Rheumatologist with primary contact	No
Panellist 9	France	Family physician with primary contact	No
Panellist 10	France	Specialist of physical and rehabilitation medicine with primary contact	No
Panellist 11	France	Emergency physician	No

**Table 2:** Relevance and formulation of items in the model in rounds 1 and 2.

	Round 1		Round 2
Items/Category	Relevance	Formulation	Relevance and Formulation
Trauma	(5.5, 4.5) ^V^	(5, 3.25) ^V^	(6, 2) ^V^
History	(2.5, 1.75) ^NV^	(3.5, 4.5) ^D^	-
Anamnesis	(5.5, 3.5) ^V^	(6, 1.75) ^V^	-
Physical examination	(6, 2) ^V^	(6.5, 1.75) ^V^	-
Fracture	(4.5, 2.5) ^D^	(5.5, 2.5) ^V^	(5.5, 1.5) ^V^
Cancer	(2.5, 4) ^NV^	(6, 4) ^V^	(4.5, 2.5) ^D^
Infection	(4, 2.25) ^D^	(3.5, 3.5) ^D^	(4.3, 3.25) ^D^
Ankylosing spondylitis	(3.5, 1.5) ^D^	(2.5, 4.25) ^NV^	(6, 1.75) ^V^
Cauda equina syndrome	(4, 2.75) ^D^	(5.5, 4.25) ^V^	(5.5, 2) ^V^
Yellow, blue and black flags	(2.5, 5.25) ^NV^	(4.5, 4.25) ^D^	(5, 4) ^V^
Treatment	(2, 2) ^NV^	(5, 4.5) ^V^	-
Additional investigations	(2, 2) ^NV^	(4.5, 4.5) ^D^	-
Favourable patient evolution	(3, 2) ^D^	(4, 5) ^D^	-
Level of preoccupation and decision	-	-	(5, 2.25) ^V^

(Median, interquartile); ^V^: item validated; ^D^: item subject to discussion; ^NV^: item not validated.

**Table 3:** Evaluation of clinical reasoning in the model.

	Round 1	Round 2
Items/Category	Evaluation	
Clinical reasoning	(3.5, 2.75) ^D^	(4, 3.25) ^D^

(Median, interquartile); ^D^: item subject to discussion.

**Table 4:** Evaluation of clinical reasoning in the model.

	Clinical Scenario n°1	Clinical Scenario n°2	Clinical Scenario n°3	Clinical Scenario n°4	Clinical Scenario n°5
Level of concern	High (5/5) *	High (3/5) No level of concern (2/5)	High (3/5) Moderate (2/5)	High (3/5) Moderate (2/5)	Moderate (3/5) No level of concern (2/5)
Important clinical findings	Age (4/5) A context of fall/trauma (5/5) Being menopausal (5/5) Being a smoker and a female (3/5)	Age (2/5) Night pain (3/5), The efficacy of NSAI drugs (2/5) The presence of enthesitis (2/5)	History of cancer (5/5) Night pain Worsening of pain Thoracic irradiation (3/5)	Weird sensation when the patient urinates (5/5) Episodes of numbness and tingling (4/5) and presence of neurological disorders in both legs (3/5)	No red flags, many blue and yellow flags (3/5) Sedentary, stressed, divorced and presence of tingling and pins and needles (2/5)
Most probable diagnoses and percentages of certainty	Fracture (5/5): 40–90%	AS (3/5), 50–85% CNSLBP (1/5): 70% MLBP (1/5): 100%	Cancer (4/5): 50–70% NSLBP (1/5): 40%	CES (4/5): 30–70% MLBP (1/5): 50%	NSLBP: 66–80% (4/5) NSLBP with radicular symptoms: 100% (1/5)
Panellist statement **	Did not have enough clinical elements (4/5) Had sufficient clinical elements (1/5)	Had sufficient clinical elements (4/5) Did not have enough clinical evidence (1/5)	Did not have enough clinical elements (4/5) Had sufficient clinical elements (1/5 NSLBP proposed)	Did not have enough clinical elements (4/5) Had sufficient clinical elements (1/5)	Did not have enough clinical elements (3/5) Had sufficient clinical elements (2/5)

Hx: history; AS: ankylosing spondyloarthritis; NSAIs: nonsteroidal anti-inflammatories; CNSLBP: chronic nonspecific low back pain; NSLBP: nonspecific low back pain; MLBP: mechanical low back pain; CES: cauda equina syndrome; n/5 number of panellists in agreement; * 2 panellists proposed AS diagnosis and 2 panellists proposed CNSLBP and MLB; ** see Appendix D for more details.

## Data Availability

All the raw data and materials are available from the authors on request.

## Appendix A

For red-flag elements:

	From PubMed, until March 2021	
	Search equations	
#1	((low back pain[MeSH Terms]) OR (back pain injuries[MeSH Terms])) AND (triage[MeSH Terms])	12
#2	((low back pain[MeSH Terms]) AND (Diagnosis, Differential[MeSH Terms])	15
#3	((((low back pain) AND (diagnosis)) AND (red flag*)) AND (primary health care)) AND (humans)	20
#4	((((low back pain) AND (diagnosis)) AND (red flag*)) AND (humans)	60

	From Scholar, until March 2021	
	Search equations	
#1	low back pain + triage + Primary Health Care + diagnosis, differential + humans + Symptom Assessment + Cauda Equina Syndrome + infection + Fractures + spinal + Pathology + cancer + red flags	74
#2	low back pain + triage + diagnosis, differential + humans + primary health care + Symptom Assessment + Cauda Equina Syndrome + spinal + Pathology + red flags + specific spinal pathologies	90
#3	Low back pain + Primary Health Care + diagnosis, differential + humans + primary health care + Symptom Assessment + Cauda Equina Syndrome + infection + Fractures + spinal + Pathology + cancer + red flags	278

	From Cochrane and Embase, until March 2021	
	Search equations	
#1	[Low Back Pain] explode all trees and with qualifier(s): [diagnosis—DI]	105
#2	Low back pain AND Diagnosis AND Cancer AND Spinal AND Infection AND Humans	78
#3	Low back pain AND Clinical reasoning	65

For yellow-flag elements:

	From PubMed, until March 2021	
	Search equations	
#1	(low back pain [Mesh] OR low back pain [tiab]) AND (yellow flag* OR psychological risk factor* OR biopsychosocial OR psychosocial) AND (screening OR evaluation OR diagnosis) AND (prognosis OR predictors)	129
#2	(low back pain low [Mesh] or low back pain [tiab]) AND (yellow flag* OR psychological risk factor* OR biopsychosocial)	251

	From Scholar, until March 2021	
	Search equations	
#1	“low back pain” (“psychological risk factor” OR “yellow flag”)	303

	For Cochrane and Embase, until March 2021	
	Search equations	
#1	(yellow flag AND psychological risk factor OR biopsychosocial) AND low back pain	463

## Appendix B

https://osf.io/fzx82/?view_only=6fd7c26d95664fafab6adb224d1fc7ff, (accessed on 9 July 2023).

## Appendix C

- Clinical scenario n°1:

A 65-year-old patient sees you for low back pain. According to the patient, this pain appeared three days ago following a nasty fall. She explains that she slipped on a patch of ice while getting to work. She reports that she suddenly fell on her buttocks. In addition, she states that she felt a sharp pain at that moment. However, she could go painfully to the place of her work. Once there, she felt a substantial pain at the top of her right buttock. It was particularly marked while sitting; she stood up regularly on the first day to relieve the ache. The pain at the top of her right buttock is constant and does not give way. The patient is not able to assign a score to her pain. She has not perceived any change for three days. She mentioned that she used a memory foam cushion to try to reduce her pain, but it did not relieve her. In addition, she says she has taken painkillers within the last three days, but they have not had any effect. Since this episode, the patient has had much trouble falling asleep. She sleeps five hours a night (usually, she sleeps about seven hours). According to her, sleeping is impossible if the buttocks are bearing weight either on the side or the back, so she can only sleep on her stomach. The patient’s history indicates that she was diagnosed with celiac disease when she was five years old, went through early menopause at thirty-nine and is a former smoker (stopped five months ago, fifteen cigarettes a day for fifteen years). As the last point, the patient reports a bruise at the top of the right buttock.

Although she has easily been bruised, she thinks this bruise did not exist before the fall. No neurological disorder was identified, and the morphostatic examination did not identify any disorders. The patient is worried because she is afraid of having broken something. She is visiting you because her family doctor cannot see her. In addition, as a chartered accountant, it is a pivotal period with a massive amount of work. Due to this pain, she fell far behind in delivering her calendar-year-end reports. From a sporting point of view, she would like to be able to resume her activities, i.e., dance classes twice a week.

- Clinical scenario n°2:

A 27-year-old patient consults you for low back pain that has been present for five days. The patient does not know what could have caused this pain. He is currently in the last year of his thesis in biochemistry, and he spends a long time sitting, but he says that he also does a lot of physical activities, such as running and cross-fit (three to five times a week). In addition, he describes another pain in the left heel that can sometimes hinder him from running. He does not remember how long it has bothered him. He manages to maintain his physical activities except during severe episodes. He specifies that he has often had periods of low back pain since he was 20 years old. He associates these periods either with his sitting posture or stress due to professional constraints or sporting overactivity. In addition, he has noticed that his sleep is often impaired during painful episodes and that he has some trouble going back to sleep once awake. The patient has a history of a sprain in the right ankle in 2017, a fracture of the right shoulder following a fall from a scooter in 2012 and psoriasis diagnosed in 2020. Referring to this painful episode, the patient has no notion of trauma. The physical examination did not identify any neurological disorders; no disorders were identified at the cutaneous and morphostatic levels. The patient has turned to you because he would eventually like to understand what is happening to him. He mentions that in painful episodes, he was prescribed nonsteroidal anti-inflammatory drugs (NSAIDs), which relieved him but also resulted in stomach pain. He would like to stop taking NSAIDs.

- Clinical scenario n°3:

A 54-year-old female patient consults you for lumbar pains which have occurred for about 3–4 weeks. These have gradually settled in and have gradually increased. This patient is a high school teacher, and lives in a first-floor apartment. She remembers helping her husband to unload the trunk after a family trip and asks if this is not related. The patient has had an episode of back pain in the past, but only during two 2 weeks. She describes a current pain which gradually bothers her more and more, especially at night. Moreover, she is beginning to have difficulty walking in relationship to severe pain. She locates her pain in the lumbar region extending behind the leg and to the thoracic region. She tried after two weeks of to resume her daily activities (running, painting, Nordic walking); there is no improvement. So, she stopped this process after a few days. However, she is not a fan of medications, she has taken NSAIDs; they were ineffective. The patient reports that her nights are very complicated at this moment as if the pain was stronger at night than during the day. The patient has also been complaining of stomach aches for 2 weeks. The physical examination did not identify any neurological disorder. In addition, there is nothing to report at the cutaneous and morphostatic level. The patient’s history includes sprained wrist in her youth, thyroid cancer at age 51. The thyroid has been removed; substitution therapy has been proposed. The patient is worried because she fears having a crumpled muscle or a muscle tear. She does not understand why her pain persists. She also consulted a masseur in order to try to relieve it. She came out relaxed, but her pain was still present.

- Clinical scenario n°4:

A 40-year-old patient comes to see you following pain in the lower back. This appeared two days ago on their way home from a jog. There is no context of any trauma. The patient specifies that he initially had pain in the left leg, and since this morning, he has had pain in both legs. He thinks they are muscular aches but more intense than usual. The patient’s situation is as follows: he is single and works as a teacher in a management school. The patient is a regular runner and reports that he is in preparation for a future half marathon, which takes place in 2 months. In addition, he sleeps less than usual; he mentions that he sleeps 5 h instead of 7 h. For one week, the patient has had stomach aches and episodes of numbness and tingling in the thighs; then, he reports feeling weird when he urinates. He has some difficulty describing the last symptom. No neurological disorder is identified at the physical examination, and there is nothing to report at the cutaneous and morphostatic levels. The patient’s history refers to tendinopathy of the right Achilles tendon four years ago and a sprain on the right ankle two years ago. The patient wants to know if this pain will go away independently or if he should start any special treatment.

- Clinical scenario n°5:

A 44-year-old patient comes to see you for back pain that appeared six days ago. He woke up one morning with a bar on his back. That helm feeling is always present and has disabled him in his daily activities. In a sitting position, he tells you that he has the sensation of having paraesthesia/tingling in his left leg. The patient is a trader, currently in divorce proceedings with his ex-wife. In addition, he spends about 10 to 12 h sitting with little break due to professional activities. He does not practice any physical activities because he does not have time because of his schedule. Since the onset of symptoms, he has tolerated the sitting position very poorly. He succeeds in decreasing symptoms by walking for more than 15 min, but the pain quickly comes back, accompanied by a slight improvement in paraesthesia/tingling. He also struggles with everyday tasks, such as donning pants and socks. If he acts too suddenly, it triggers severe pain in the back. The patient talks with you about his medical history: type 1 diabetes and rupture of the right anterior. The patient would like the situation to improve as soon as possible because it prevents him from fully dedicating himself to his work. Moreover, the patient mentions that the atmosphere at his work is not so good since the results of different traders are not very good as a result of various bad choices in recent investments. He is willing to actively participate in treatment but wants it to remain feasible in view of his situation.

## Appendix D

	**Clinical Scenario n°1**	**Clinical Scenario n°2**	**Clinical Scenario n°3**	**Clinical Scenario n°4**	**Clinical Scenario n°5**
Panellists’ statements	Did not have enough clinical elements (4/5), need more information about Hx of fracture and additional examinations Had sufficient clinical elements (1/5)	Had sufficient clinical elements (4/5), need more information about Hx of AS and morning stiffness (5/5) Did not have enough clinical evidence: this panellist expected blood tests, Hx of As and complementary tests (1/5)	Did not have enough clinical elements (4/5), need more information about weight loss and gait disturbance, thorough exploration of nocturnal pain and further examination Had sufficient clinical elements (1/5 NSLBP proposed)	Did not have enough clinical elements (4/5), need more information about different sensations when urinating or defecating and alteration in sexual intercourse; one talked about performing a nervous system examination More information on lower-extremity symptoms to distinguish radicular pain versus referred visceral pain (1/5 MLBP proposed) Had sufficient clinical elements (1/5), specified his wish to carry out a thorough neurological examination with MRI, EMG	Did not have enough clinical elements (3/5), would like to perform a complete clinical examination and neurological examination to detect possible disorders Had sufficient clinical elements (2/5)

## References

[1] Demont A., Bourmaud A., Kechichian A., Desmeules F. (2021). The Impact of Direct Access Physiotherapy Compared to Primary Care Physician Led Usual Care for Patients with Musculoskeletal Disorders: A Systematic Review of the Literature. Disabil. Rehabil..

[2] Hon S., Ritter R., Allen D.D. (2021). Cost-Effectiveness and Outcomes of Direct Access to Physical Therapy for Musculoskeletal Disorders Compared to Physician-First Access in the United States: Systematic Review and Meta-Analysis. Phys. Ther..

[3] Kechichian A., Desmeules F., Girard P., Pinsault N. (2022). Acceptability of a Task Sharing and Shifting Model between Family Physicians and Physiotherapists in French Multidisciplinary Primary Healthcare Centres: A Cross-Sectional Survey. Fam. Med. Community Health.

[4] Finucane L.M., Downie A., Mercer C., Greenhalgh S.M., Boissonnault W.G., Pool-Goudzwaard A.L., Beneciuk J.M., Leech R.L., Selfe J. (2020). International Framework for Red Flags for Potential Serious Spinal Pathologies. J. Orthop. Sports Phys. Ther..

[5] Galliker G., Scherer D.E., Trippolini M.A., Rasmussen-Barr E., LoMartire R., Wertli M.M. (2020). Low Back Pain in the Emergency Department: Prevalence of Serious Spinal Pathologies and Diagnostic Accuracy of Red Flags. Am. J. Med..

[6] Bardin L.D., King P., Maher C.G. (2017). Diagnostic Triage for Low Back Pain: A Practical Approach for Primary Care. Med. J. Aust..

[7] Traeger A., Buchbinder R., Harris I., Maher C. (2017). Diagnosis and Management of Low-Back Pain in Primary Care. CMAJ.

[8] Henschke N., Maher C.G., Refshauge K.M., Herbert R.D., Cumming R.G., Bleasel J., York J., Das A., McAuley J.H. (2009). Prevalence of and Screening for Serious Spinal Pathology in Patients Presenting to Primary Care Settings with Acute Low Back Pain. Arthritis Rheum..

[9] National Health Service Did You Know—Cauda Equina Syndrome. https://webar-chive.nationalarchives.gov.uk/20180903112728/.

[10] Downie F., McRitchie C., Monteith W., Turner H. (2019). Physiotherapist as an Alternative to a GP for Musculoskeletal Conditions. Br. J. Gen. Pract..

[11] Matifat E., Méquignon M., Cunningham C., Blake C., Fennelly O., Desmeules F. (2019). Benefits of Musculoskeletal Physical Therapy in Emergency Departments: A Systematic Review. Phys. Ther..

[12] Oliveira C.B., Maher C.G., Pinto R.Z., Traeger A.C., Lin C.W.C., Chenot J.F., van Tulder M., Koes B.W. (2018). Clinical Practice Guidelines for the Management of Non-Specific Low Back Pain in Primary Care: An Updated Overview. Eur. Spine J..

[13] Parreira P.C.S., Maher C.G., Traeger A.C., Hancock M.J., Downie A., Koes B.W., Ferreira M.L. (2019). Evaluation of Guideline-Endorsed Red Flags to Screen for Fracture in Patients Presenting with Low Back Pain. Br. J. Sports Med..

[14] Verhagen A.P., Downie A., Maher C.G., Koes B.W. (2017). Most Red Flags for Malignancy in Low Back Pain Guidelines Lack Empirical Support: A Systematic Review. Pain.

[15] Cook C.E., George S.Z., Reiman M.P. (2018). Red Flag Screening for Low Back Pain: Nothing to See Here, Move along: A Narrative Review. Br. J. Sports Med..

[16] Ferreira G.E., Machado G.C., Oliver M., Maher C.G. (2018). Limited Evidence for Screening for Serious Pathologies Using Red Flags in Patients with Low Back Pain Presenting to the Emergency Department. EMA-Emerg. Med. Australas..

[17] Nathalie D., Abiodun A., Dena K., Nitin T., Laura F., Lenerdene L., David M.W., Jackie S. (2019). What Is the Diagnostic Accuracy of Red Flags Related to Cauda Equina Syndrome (CES), When Compared to Magnetic Resonance Imaging (MRI)? A Systematic Review. Musculoskelet. Sci. Pract..

[18] Yusuf M., Finucane L., Selfe J. (2019). Red Flags for the Early Detection of Spinal Infection in Back Pain Patients. BMC Musculoskelet. Disord..

[19] Diamond I.R., Grant R.C., Feldman B.M., Pencharz P.B., Ling S.C., Moore A.M., Wales P.W. (2014). Defining Consensus: A Systematic Review Recommends Methodologic Criteria for Reporting of Delphi Studies. J. Clin. Epidemiol..

[20] Waggoner J., Carline J.D., Durning S.J. (2016). Is There a Consensus on Consensus Methodology? Descriptions and Recommendations for Future Consensus Research. Acad. Med..

[21] Belton I., MacDonald A., Wright G., Hamlin I. (2019). Improving the Practical Application of the Delphi Method in Group-Based Judgment: A Six-Step Prescription for a Well-Founded and Defensible Process. Technol. Forecast. Soc. Chang..

[22] Baker J., Lovell K., Harris N. (2006). How Expert Are the Experts? An Exploration of the Concept of ‘Expert’ within Delphi Panel Techniques. Nurse Res..

[23] Trevelyan E.G., Robinson N. (2015). Delphi Methodology in Health Research: How to Do It?. Eur. J. Integr. Med..

[24] Verhagen A.P., Downie A., Popal N., Maher C., Koes B.W. (2016). Red Flags Presented in Current Low Back Pain Guidelines: A Review. Eur. Spine J..

[25] Premkumar A., Godfrey W., Gottschalk M.B., Boden S.D. (2018). Red Flags for Low Back Pain Are Not Always Really Red: A Prospective Evaluation of the Clinical Utility of Commonly Used Screening Questions for Low Back Pain. J. Bone Jt. Surg. Am..

[26] Shaw B., Kinsella R., Henschke N., Walby A., Cowan S. (2020). Back Pain “Red Flags”: Which Are Most Predictive of Serious Pathology in the Emergency Department?. Eur. Spine J..

[27] Jenkins H.J., Downie A.S., Maher C.G., Moloney N.A., Magnussen J.S., Hancock M.J. (2018). Imaging for Low Back Pain: Is Clinical Use Consistent with Guidelines? A Systematic Review and Meta-Analysis. Spine J..

[28] Lin I., Wiles L., Waller R., Goucke R., Nagree Y., Gibberd M., Straker L., Maher C.G., O’Sullivan P.P.B. (2020). What Does Best Practice Care for Musculoskeletal Pain Look like? Eleven Consistent Recommendations from High-Quality Clinical Practice Guidelines: Systematic Review. Br. J. Sports Med..

[29] Mescouto K., Olson R.E., Hodges P.W., Setchell J. (2022). A Critical Review of the Biopsychosocial Model of Low Back Pain Care: Time for a New Approach?. Disabil. Rehabil..

[30] Haslam S.A., Haslam C., Jetten J., Cruwys T., Bentley S. (2019). Group Life Shapes the Psychology and Biology of Health: The Case for a Sociopsychobio Model. Soc. Personal. Psychol. Compass.

[31] Stilwell P., Harman K. (2019). An Enactive Approach to Pain: Beyond the Biopsychosocial Model. Phenomenol. Cogn. Sci..

[32] Cormack B., Stilwell P., Coninx S., Gibson J. (2022). The Biopsychosocial Model Is Lost in Translation: From Misrepresentation to an Enactive Modernization. Physiother. Theory Pract..

[33] Zhu G.C., Böttger K., Slater H., Cook C., Farrell S.F., Hailey L., Tampin B., Schmid A.B. (2019). Concurrent Validity of a Low-Cost and Time-Efficient Clinical Sensory Test Battery to Evaluate Somatosensory Dysfunction. Eur. J. Pain.

[34] Schmid A.B., Fundaun J., Tampin B. (2020). Entrapment Neuropathies: A Contemporary Approach to Pathophysiology, Clinical Assessment, and Management. PAIN Rep..

[35] Schmid A.B., Hailey L., Tampin B. (2018). Entrapment Neuropathies: Challenging Common Beliefs With Novel Evidence. J. Orthop. Sports Phys. Ther..

[36] Schmid A.B., Nee R.J., Coppieters M.W. (2013). Reappraising Entrapment Neuropathies-Mechanisms, Diagnosis and Management. Man. Ther..

[37] Bender C., Dove L., Schmid A.B. (2022). Does Your Bedside Neurological Examination for Suspected Peripheral Neuropathies Measure Up?. J. Orthop. Sports Phys. Ther..

[38] Brun C., Akinyemi A., Houtin L., Zerhouni O., Monvoisin R., Pinsault N. (2022). Intolerance of Uncertainty and Attitudes towards Vaccination Impact Vaccinal Decision While Perceived Uncertainty Does Not. Vaccines.

[39] Brun C., Zerhouni O., Akinyemi A., Houtin L., Monvoisin R., Pinsault N. (2022). Impact of Uncertainty Intolerance on Clinical Reasoning: A Scoping Review of the 21st-Century Literature. J. Eval. Clin. Pract..

